# Activity to Breast Cancer Cell Lines of Different Malignancy and Predicted Interaction with Protein Kinase C Isoforms of Royleanones

**DOI:** 10.3390/ijms21103671

**Published:** 2020-05-23

**Authors:** Vera M. S. Isca, Milan Sencanski, Nenad Filipovic, Daniel J. V. A. Dos Santos, Ana Čipak Gašparović, Lucília Saraíva, Carlos A. M. Afonso, Patrícia Rijo, Alfonso T. García-Sosa

**Affiliations:** 1 Center for Research in Biosciences & Health Technologies (CBIOS), Universidade Lusófona de Humanidades e Tecnologias, 1749-024 Lisboa, Portugal; Veraisca@msn.com; 2 Instituto de Investigação do Medicamento (iMed.ULisboa), Faculdade de Farmácia, Universidade de Lisboa, 1649-003 Lisboa, Portugal; dos.santos.daniel@gmail.com (D.J.V.A.D.S.); carlosafonso@ff.ulisboa.pt (C.A.M.A.); 3 Laboratory for Bioinformatics and Computational Chemistry, Institute of Nuclear Sciences VINCA, University of Belgrade, P.O. Box 522, 11001 Belgrade, Serbia; sencanski@vin.bg.ac.rs; 4 Department of Chemistry and Biochemistry, Faculty of Agriculture, University of Belgrade, 11001 Belgrade, Serbia; nenadf.chem@gmail.com; 5 Rudjer Boskovic Institute, Bijenicka 54, HR-10000 Zagreb, Croatia; Ana.Cipak@irb.hr; 6 LAQV/REQUIMTE, Laboratório de Microbiologia, Departamento de Ciências Biológicas, Faculdade de Farmácia, Universidade do Porto, Rua de Jorge Viterbo Ferreira n.º 228, 4050-313 Porto, Portugal; lucilia.saraiva@ff.up.pt; 7 Institute of Chemistry, University of Tartu, Ravila 14a, 54011 Tartu, Estonia

**Keywords:** *Plectranthus*, royleanones, hemi-synthesis, PKC activity, isozyme-selectivity, molecular interactions

## Abstract

Plants have been used for centuries to treat several illnesses. The *Plectranthus* genus has a vast variety of species that has allowed the isolation of cytotoxic compounds with notable activities. The abietane diterpenes 6,7-dehydroroyleanone (DeRoy, **1**), 7α-acetoxy-6β-hydroxyroyleanone (Roy, **2**), and Parvifloron D (ParvD, **3**) were obtained from *Plectranthus* spp. and showed promising biological activities, such as cytotoxicity. The inhibitory effects of the different natural abietanes (**1**-**3**) were compared in MFC7, SkBr3, and SUM159 cell lines, as well as SUM159 grown in cancer stem cell-inducing conditions. Based on the royleanones’ bioactivity, the derivatives RoyBz (**4**), RoyBzCl (**5**), RoyPr_2_ (**6**), and DihydroxyRoy (**7**), previously obtained from **2**, were selected for further studies. Protein kinases C (PKCs) are involved in several carcinogenic processes. Thus, PKCs are potential targets for cancer therapy. To date, the portfolio of available PKC modulators remains very limited due to the difficulty of designing isozyme-selective PKC modulators. As such, molecular docking was used to evaluate royleanones **1**-**6** as predicted isozyme-selective PKC binders. Subtle changes in the binding site of each PKC isoform change the predicted interaction profiles of the ligands. Subtle changes in royleanone substitution patterns, such as a double substitution only with non-substituted phenyls, or hydroxybenzoate at position four that flips the binding mode of ParvD (**3**), can increase the predicted interactions in certain PKC subtypes.

## 1. Introduction

Although many commercially available chemotherapeutic agents are plant-derived products (e.g., paclitaxel, vincristine, irinotecan), some may induce resistance and toxic effects that can compromise therapy success and consequent patient recovery [1]. Therefore, the search for novel effective drugs with suppressed or lower toxicity on normal cells is still demanded [1], as well as the elucidation of their effect on the modulation of cell death mechanisms and consequent affected targets, is also very important [2,3]. 

**Figure 1:**
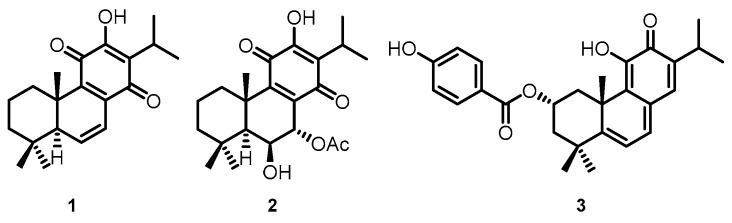
(**1**) 6,7-dehydroroyleanone (DeRoy); (**2**) 7*α*-acetoxy-6*β*-hydroxyroyleanone (Roy); and (**3**) Parvifloron D (ParvD) isolated from *Plectranthus* spp.

The *Plectranthus* genus has a vast variety of species scattered throughout tropical Africa, Asia, and Australia, and is known for its broad spectrum of activities [4,5]. Several species have already been screened for the presence of cytotoxic compounds with notable activities [3,6,7], such as secondary metabolites of diterpenes belonging to the abietane class [8,9,10]. Royleanones are bioactive compounds with a tricyclic skeleton characteristic of the abietane class and can possess phenolic and quinonic moieties [11]. 6,7-dehydroroyleanone (DeRoy, **1**, Figure 1) was identified as the main component of the *P. madagascariensis* (Pers.) Benth essential oil [12]. DeRoy (**1**) showed promising antioxidant, antimicrobial, and cytotoxic activity [3,13]. In the same way, the compound 7*α*-acetoxy-6*β*-hydroxyroyleanone (Roy, **2**, Figure 1) obtained from *P. grandidentatus* exhibited cytotoxicity at low concentrations against different types of human cancer cell lines (MCF-7, NCI-H460, SF-268, TK-10, UACC-62) [9]. Parvifloron D (ParvD, **3**, Figure 1) is the main phytochemical constituent of *P. ecklonii* with cytotoxic activity against several human tumor cells and an apoptotic inducer in leukemia cells. [14].

**Figure 2:**
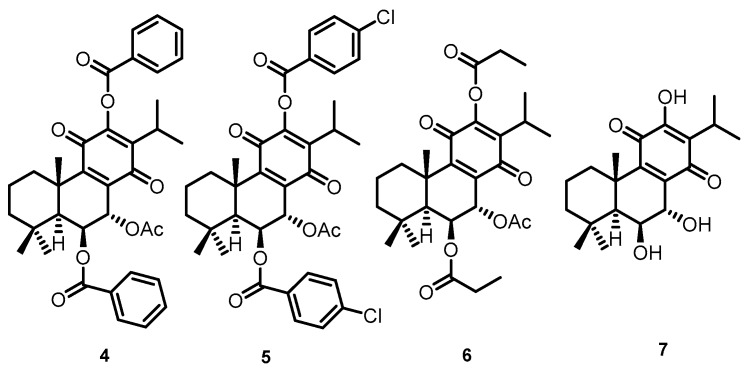
Chemical structures of semi-synthetic derivatives: (**4**) RoyBz, (**5**) RoyBzCl, (**6**) RoyPr_2_, and (**7**) DihydroxyRoy.

These natural royleanones can be interesting leads for drug development by enhancing cytotoxic properties. They contain acidic hydroxyl groups suitable for derivatization. Furthermore, the patented diterpene 6*β*-benzoyloxy-12-*O*-benzoylroyleanone (RoyBz, **4**, Figure 2) is the first reported protein kinase C*δ* (PKC*δ*) selective activator [15] and was obtained by semi-synthesis from compound **2** [16]. In fact, a recent study reported that **4** potently inhibits the proliferation of colon cancer cells through a PKC*δ*-dependent mitochondrial apoptotic pathway involving caspase-3 activation [15]. These results have suggested that abietanes can originate a new class of PKC*δ*-selective modulators with potential application for colon cancer therapy. Other interesting derivatives prepared using **2** as a starting material are 7*α*-acetoxy-6*β*-(4-chloro) benzoyloxy-12-*O*-(4-chloro)benzoylroyleanone (RoyBzCl, **5**), 7*α*-acetoxy-6*β*-propanoyloxi-12-*O*-propanoylroyleanone (RoyPr_2_, **6**), and 6,7-dihydroxyroyleanone (DihydroxyRoy, **7**) (Figure 2). Nonetheless, to date, the effect of **4**, **5**, and **6** as PKC modulators has not been investigated.

PKC is an attractive target for cancer therapy as recently demonstrated by pre-clinical and clinical data [17]. PKC is a large family of serine-threonine kinases whose isozymes share a conserved N-terminal regulatory region that comprises C1 and C2 domains, and a C-terminal catalytic region responsible for ATP binding and phosphotransferase activity [17,18]. To date, 10 isozymes of PKC have been identified and can be grouped into three subfamilies: Classical (*α*, *β*I, *β*II, and *γ*), novel (*δ*, *ε*, *η*, and *θ*), and atypical PKCs (*ζ* and *λ*\*ι*) [17,19]. These isozymes differ in structure, function, and biochemical properties. The PKC family has been intimately associated with the development and progression of cancer and plays a major role in numerous metabolic and signaling pathways associated with proliferation, migration, differentiation, apoptosis, invasion, tumorigenesis, and metastasis. Therefore, finding new clinically effective modulators of their activity becomes a priority when targeting cancer treatment [15]. 

The major challenge from a pharmacological standpoint for the design of isozyme-selective PKC modulators is that PKCs are highly related among them, as well as to other structurally related kinases [20,21]. Furthermore, PKC isozymes may exhibit overlapping as well as opposite functions in cancer development [21]. PKC*α* seems to be related to malignant glioma cells’ growth [22]. PKC*β* isoforms have been associated with progression of breast cancer [23], colon cancer [24], and prostate cancer [25]. Moreover, PKC*ι* is implicated in the progression of lung cancer [26] and ovarian tumor [27]. An overexpression of PKC*ε* was observed in head and neck squamous cell carcinoma [28], and human non-small cell lung cancer [29]. Increased activity of PKC*ζ* was found to promote tumorigenesis in breast [30] and pancreatic [31] cancer. PKC*ζ* also seems to function as a tumor suppressor in prostate cancer [32].

Human pluripotent stem (hPS) cells possess the ability of self-renewal, a process heavily supported by the existence of signaling pathways such as those of PKC, among others [33]. As such, cancer stem cells (CSCs) have tumor-initiating properties and have been identified in a variety of carcinomas including breast cancer [34]. In fact, these are responsible for cancer-related phenomena such as tumor growth, recurrence, and metastasis [34]. Additionally, although some chemicals or radiations are effective in eliminating the bulk of cancer cells, the majority fail to eliminate the CSC subpopulation leading to the generation of new tumors [34]. Therefore, recent research has focused on finding effective treatments targeting these cells. PKC subtypes such as PKC*α* are a central target when addressing toxicity for breast cancer stem cells and their expression is usually associated with aggressive triple-negative breast cancers. In this way, their inhibition is crucial when targeting breast CSCs [34]. 

PKCs are activated by endogenous calcium, diacylglycerol, phospholipids, and/or tumor-promoting phorbol esters, binding in the C1 domain. Other structurally diverse activators isolated from different organisms have been described [35]. It is generally accepted that most of the PKC activators bind to the regulatory domain [17,35]. On the other hand, kinase inhibitors are ATP-competitive small molecules that bind to the kinase domain (ATP binding site) of the PKC isozymes [36]. Currently, several classes of PKC inhibitors exist at different levels of development and some of them have reached clinical trials for the treatment of different cancers, such as bryostatin 1, curcumin, staurosporine, midostaurin, and sotrastaurin [35,36]. Unfortunately, to date, the portfolio of available PKC modulators remains very limited and most of the small molecules identified lack specificity among isoforms of the PKC family or even with other kinases unrelated to PKC, which is not suitable for clinical use [17]. Due to this, the search for more selective and potent ATP-competitive inhibitors remains a promising strategy for the future of anticancer treatment. 

To our knowledge, royleanones have never been evaluated as PKC inhibitors. Herein, molecular docking studies were performed for compounds **1**-**6** in order to clarify the molecular interaction to the kinase domain of PKC isoforms. Molecular docking techniques are valuable because they can provide initial information on possible binding sites, binding energies, and specific ligand–residue interactions. These results may help in the selection of which derivatives should be synthesized for structure–activity relationships in the different PKC isoforms, also providing clues towards their specificity and design. The activity towards breast cancer cell lines of different malignancy for the natural abietanes **1** to **3** isolated from *Plectranthus* spp. was also evaluated in vitro. 

## 2. Results

### 2.1. MTT Breast Cancer

The inhibitory effects of the different natural royleanones (**1**-**3**) were compared in MFC-7, SkBr3, SUM159, and SUM159 grown in CSC-inducing conditions. CSC represents the subpopulation of cancer cells that is responsible for metastasis. Due to their morphological characteristics of growth in spheres, they are referred to as SUM159 spheres. Three-dimensional in vitro models can be considered as an intermediate model between in vitro cancer cell line cultures and in vivo tumor. Different cancer cell lines were used aiming to assess which of the cell development phases is most affected by the action of each abietane compound.

**Figure 3:**
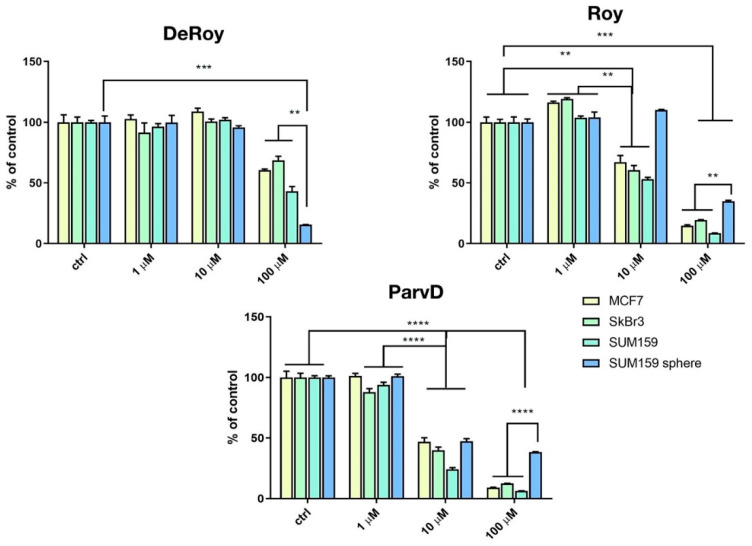
Cytotoxicity activity (MTT assay) on MCF-7 (hormone-positive breast cancer cells), SkBr3 Her-positive, SUM159 triple-negative, and SUM159 spheres. Statistical significance between marked groups: ** *p* < 0.01, *** *p* < 0.001, **** *p* < 0.0001.

The results showed that increased concentrations of abietanes reduced cell viability (Figure 3). Overall, although Roy (**2**) and ParvD (**3**) tend to inhibit MCF-7, SkBr3, and SUM159 cell viability, it was shown to be less effective against the most aggressive type of cells, the cancer stem cells SUM159 sphere. DeRoy (**1**) showed the highest inhibitory effect on SUM159 spheres, thus indicating its potential to significantly decrease the number of viable CSC cells. 

### 2.2. Docking Results

The Protein DataBank (PDB, [37]) had the following available protein crystal structures: *θ*: 5F9E; *ι*: 3ZH8, 3AW8, 3A8X (only 3ZH8 has a co-crystallized inhibitor); *α*: 4RA4; and *β*II: 2I0E. There are crystal structures of other isomorphs; however, structures 1YRK and 2YUU (*δ*), and 2WH0 (*ε*) do not contain the catalytic domain, while no structure of the *ζ* isoform is available. From the percent identity matrix (Table 1), isomorph *θ* seems to be the most similar to isoform *δ*. Based on these results, we took structures 4RA4 for isoform *α*, 3ZH8 for isoform *ι* as an approximation to *ζ*, and 5F9E for isoform *θ* (and as an approximation to isoform *δ*). Isoform *α* was taken as an approximation to isoform *ε*. 1PTR was taken for isoform *δ*.

The results from the docking runs of all programs, proteins, and ligands (Table 2) showed that ParvD (**3**), Roy (**2**), and DeRoy (**1**) tended to have calculated docking scores close to those of the co-crystallized ligands (i.e., known binders) for all five isoforms studied. The MMGBSA results only showed a favorable calculated docking scores for **1** in isoforms *θ*, *β*, and *ι* but not in *α*. These results for the interaction can be compared to experimental observations showing that compounds **3** and **1** are the strongest inhibitors for all cell types. The weakest docking scores correspond to the compounds docked into the PKCδ isoform. 

**Figure 4:**
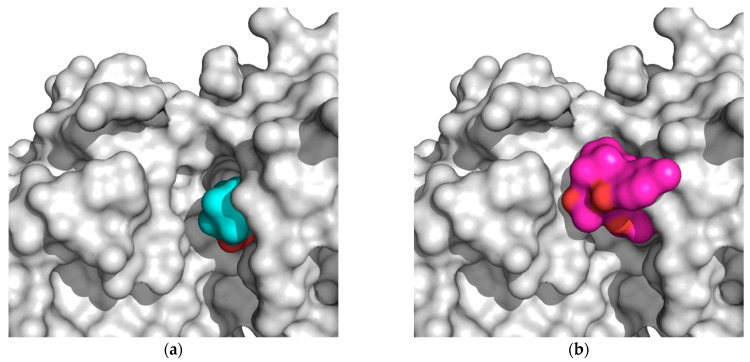
(**a**) Deeply bound and less exposed conformation of DeRoy (**1**, cyan) in PKC (white); (**b**) more exposed bound conformation of RoyBzCl (**5**, magenta) in PKC (white). All molecules in solvent-accessible surface area representation.

From the data presented in Table 1, Table 2, Table 3, Table 4 and Table 5, we can explain the general trends in the docking scores of royleanones to different isoforms of PKC. Docking calculations report docking scores of up to −9.9 kcal/mol for **1** and **3**. From the intermolecular interaction analysis (Table 3), **1** interacts with 11 amino acid residues in a hydrophobic manner. Kinases share overlapping substrate recognition patterns but specific hydrophobic binding pockets for recognizing bulky hydrophobic residues upstream of the catalytic Ser/Thr distinguish atypical PKCs from other kinases, and this may provide specificity. In addition, although reportedly forming a hydrophobic interaction with the conserved Lys 371, we cannot rule out the possibility for hydrogen bond formation as well, analogous to the case in the *α* isoform (with a corresponding conserved residue Lys 368). This actually is significantly different to compound **2**, as **1** is capable of forming this additional interaction. Moreover, a significant difference in the log*P* values (Table 5) between **2** and **1** makes the latter more favorable for the local hydrophilic environment of the binding site (Thr 404, Lys 371, Met 473, and Met 420, if considered as amphipathic), rather than the more lipophilic **2**. In addition, if we compare the percent of exposed surface area of the docked conformations in the PKC *α* isoform, **1** is more “buried”, and therefore more prone to keeping the bound conformation than **2**. Regarding **5** and **6**, both have similar binding patterns to compounds **2** and **1**. However, the Autodock docking score values are contradictory with respect to the experimental inhibition results. Therefore, the explanation may be found in the compounds’ properties. **5** has a similar log*P* value and ratio of the exposed surface as **1**, while **5** has the highest log*P* value and a more exposed surface. This is probably the reason for the lower interaction score: The compound is too nonpolar and less buried in the binding site, which contributes to an easier unbinding process. Figure 4 shows the difference in the exposed surface area for **1** and **5**. 

Compound **3** shows the best docking score towards the PKC *β*II isoform (−9.9 kcal/mol), however, without selectivity towards other isoforms. The high predicted affinity may be explained by the formation of more hydrophobic interactions with binding site amino acids than in the case of **1** and **2**. In addition, **3** has a higher log*P* value, and the ratio between the total solvent-exposed and docked conformation solvent-exposed surface is the lowest in the case of **3,** which indicates a slower unbinding process. Both properties are in favor of a higher predicted affinity towards any of the PKC isoforms. 

The difference in docking scores between **1** and **4** can be explained by the scoring function’s overestimation of hydrophobic interactions. The docking results identified **3** and **1** as the predicted most strongly interacting compounds to all PKC isoforms, although less strongly to *δ*. Finally, the predicted interaction of each compound in different PKC isoforms can be due to subtle but significant amino acid residue changes, such as Met → Leu or Thr → Val and others, which can change the electrostatic nature of the binding site towards being more hydrophobic.

**Figure 5:**
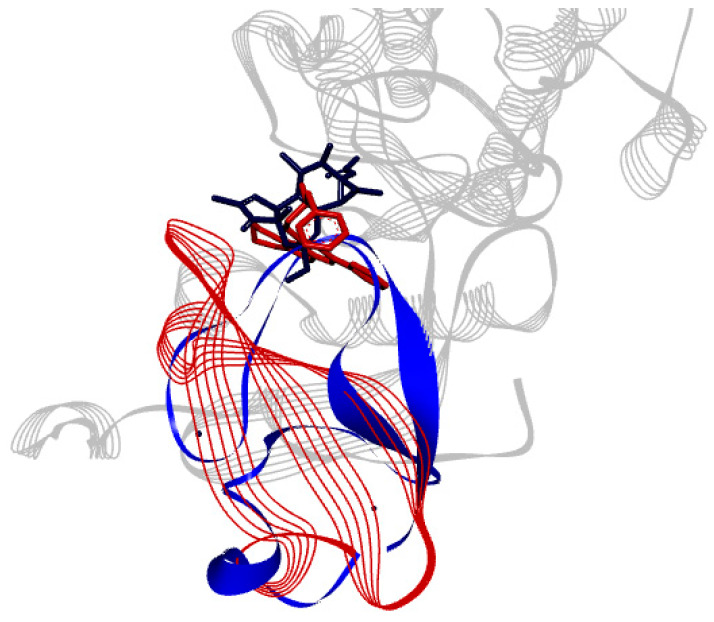
Alignment of tertiary structure of the catalytic binding sites for PKC*δ* (blue) and PKC*ι* (red).

The binding site of PKC*δ* can be seen to be aligned onto PKC*ι* (Figure 5). ParvD (**3**), DeRoy (**1**), and RoyBz (**4**) are moderately sized compounds compared to the others in this series, which may allow for a better fit in the different PKC binding sites, though PKC*δ* has a smaller binding site as compared to the other isoforms. 

**Figure 6:**
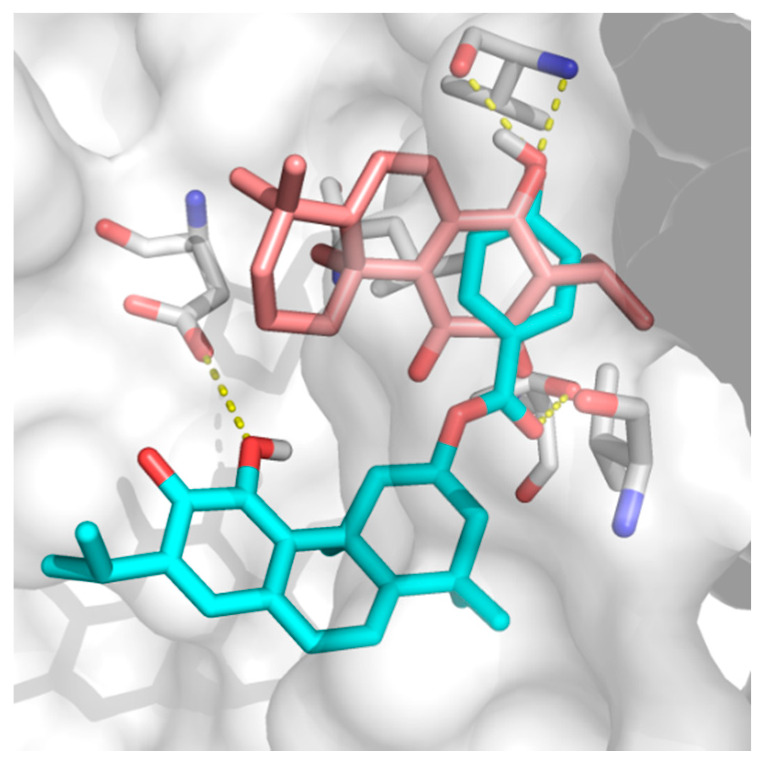
Docked binding poses of (**3**) ParvD (cyan) and (**1**) DeRoy (pink) in the binding site of PKC*ι*.

ParvD (**3**) has the best interaction profile of **1**-**3**, given that it is the most potent compound against all cells studied, including the most aggressive type. The docked binding pose of ParvD (**3**) in 3ZH8 shows a significant difference with respect to DeRoy (**1**) (Figure 6), with the royleanone part of ParvD (3) flipped and more towards the entrance to the binding site, and its phenol ring in the hydroxybenzoate functional group interacting deeper, making hydrogen bonds with Val326 (top-right in Figure 6, interactions made instead by the royleanone part in the DeRoy(**1**) complex). Table 2 also shows that the strongest predicted interaction for the strongest experimental inhibitor, ParvD (**3**), is with isoform PKC*ι*, 3ZH8, where ParvD (**3**) outscores the other compounds, including DeRoy (**1**).

**Figure 7:**
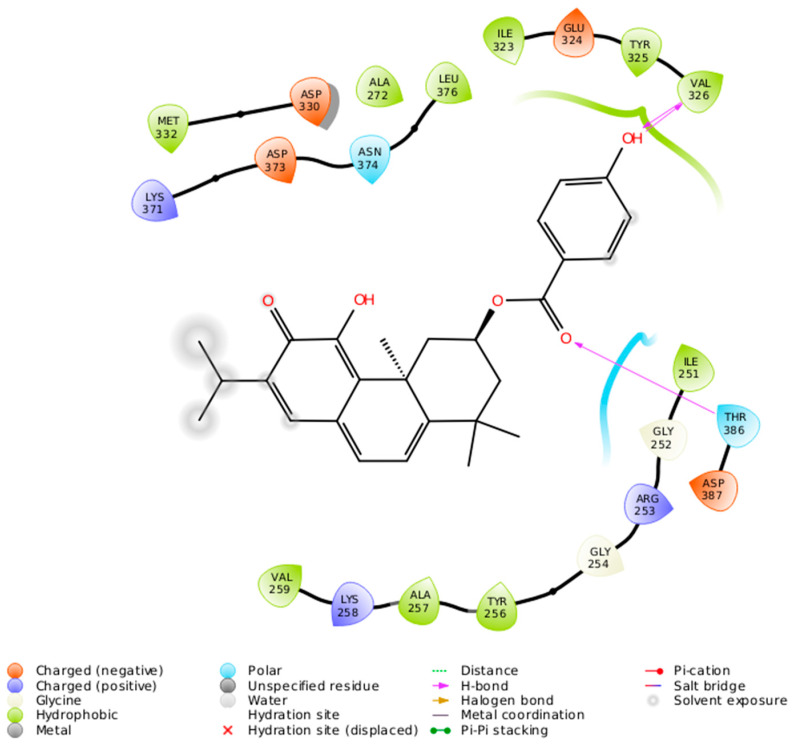
Intermolecular protein–ligand interactions for ParvD (**3**) in the binding site of PKC*ι*.

The 2-D interaction diagram (Figure 7) shows a schematic representation of the fit and intermolecular protein–ligand interactions for ParvD (**3**) and PKC*ι*.

## 3. Discussion

Even though the mechanism of roylaneone compound inhibition of cancer cells is not yet determined as exclusively through inhibition or activation (**4** activates PKC*δ* [15]) of specific PKC isoforms, ParvD (**3**) has the best calculated interaction profile of compounds **1**-**3**, given its good interactions of the core structure and substituents with Val326 in the PKC binding site, as well as experimental inhibition against all cell types, including the most aggressive form. Structure 3ZH8, representing isoform PKC*ι*, appears to best reproduce experimental trends, and therefore, may provide clues for compound design. Substitution groups, such as hydroxybenzoate on position 4 of the royleanone core, can provide a better docked binding pose. In addition, polar groups at the mouth of the binding site are in close proximity to the 1,1 dimethyl groups. A possible route for further modification may be decorating or substituting these 1,1-methyl groups with polar functional groups able to make hydrogen bonds with these residues on PKC, such as –OH or –NH_3,_ as well as improving the compounds’ log*P* value. 

Clues on PKC isoform modulation may give information on the specificity towards each isoform based on the structure of their different biding sites, as well as on useful probe compounds, such as royleanones. Even if it may be difficult to pick up differences in the binding sites of PKC isoforms, this is indeed possible. Selective thieno[2,3-*d*]pyrimidine-based chemical inhibitors of atypical PKCs have been reported [38], and the region of hydrophobic residues in the binding site upstream from the catalytic Lys/Thr provides this specificity for compounds with bulky hydrophobic groups. The conserved Lys 371, on the other hand, provides a binding partner in nearly all isoforms. Atypical PKCs can tolerate the Lys -> Trp mutation, whereas other PKCs cannot [39].

Compounds in phase I or phase II clinical trials targeting classical PKC isoforms were not successful [31], but recent studies implicate that mainly atypical and novel PKC enzymes regulate oncogenic signaling pathways in pancreatic cancer. These subgroups converge signaling induced by mutant K-Ras, inflammatory cytokines, and growth factors. Approaches to compound design for novel PKCs and atypical PKCs may include allosteric inhibitors and ATP competitive inhibitors. The royleanone core and derivatives are interesting for further research on their different interactions with different PKC isoforms, pancreatic cancer, and breast cancer cell lines with an emphasis on breast CSC, which are attractive target cells as these are the cells with the highest metastatic potential.

## 4. Materials and Methods 

### 4.1. Compounds

Compounds **1**, **2**, and **3** were isolated from *Plectranthus* spp. DeRoy (**1**) was obtained from the essential oil through the hydrodistillation of leaves and steams of *P. masdascariensis* (Pers.) Benth. in a Clevenger-apparatus, according to C. Garcia et al., 2018 [3]. Acetonic extraction and isolation of Roy (**2**) from aerial parts of *P. grandidentatus* Gürke, was adapted from the procedure described on Bernardes C.E.S. et al., 2018 [40]. ParvD (**3**) was isolated from the acetonic extract of the whole plant *P. ecklonii* Benth, according to Simões et al., 2010 [41]. On the other hand, RoyBz (**4**), RoyBzCl (**5**), RoyPr_2_ (**6**), and DihydroxyRoy (**7**) are derivatives previously obtained from **2** [42]. All compounds were used pure and their structures were confirmed by spectroscopic means and compared to literature data. 

**DeRoy (1):** Orange-red needles, **^1^H NMR** (CDCl_3_, 300 MHz, ppm): δ 7.34 (1H, s, OH-12), 6.81 (1H, dd, *J* = 9.7, 3.2 Hz, H-7), 6.46 (1H, dd, *J* = 9.7, 3.2 Hz, H-6), 3.16 (1H, hept, *J* = 7.1 Hz, H-15), 2.88 (1H, dt, *J* = 13.3 Hz, H-1β), 2.13 (1H, t, *J* = 3.2 Hz, H-5α), 1.63–1.60 (1H, t, H-2α), 1.52–1.50 (1H, t, H-2β), 1.47–1.46 (1H, m, H-3β), 1.42 (1H, d, *J* = 4.2 Hz, H-1α), 1.23 (1H, s, H-3α), 1.22 (Me-16, overlapped signal), 1.20 (Me-17, overlapped signal), 1.03 (3H, s, Me-20), 1.01(3H, s, Me-18), 0.98 (3H, s, Me-19). **^13^C NMR** (CDCl_3_, 75 MHz, ppm): δ; 186.20 (C-14), 183.58 (C-11), 151.34 (C-12), 140.64 (C-9), 139.8 (C-6), 138.6 (C-8), 122.72 (C-13), 121.33 (C-7), 52.23 (C-5), 40.64 (C-4), 39.38 (C-3), 35.28 (C-1), 33.40 (C-10), 32.74 (C-19), 24.21 (C-18), 22.94 (C-15), 20.14 (C-17), 19.95 (C-16), 18.81 (C-2), 15.31 (C-20).

**Roy (2):** Yellow quadrangular plates, **^1^H-NMR** (CDCl_3_, 300 MHz, ppm): δ 7.22 (1H, s, 12-OH), 5.66 (1H, dd, *J* = 2.2, 0.7 Hz, H-7β), 4.31 (1H, s, H-6α), 3.16 (1H, sept, *J* = 7.1 Hz, H-15), 2.63 (1H, d, *J* = 12.8 Hz, H-1β), 2.04 (3H, s, Me-7α-OAc), 1.89–1.78 (1H, m, H-2β), 1.61 (3H, s, Me-20), 1.55–1.46 (2H, m, H-2α and H-3β, overlapped signal), 1.33 (1H, s, H-5α), 1.23 (3H, s, Me-19, overlapped signal), 1.22 (3H, s, Me-17, overlapped signal), 1.21 (1H, s, H-3α, overlapped signal), 1.20 (3H, s, Me-16, overlapped signal), 1.18 (1H, s, H-1α, overlapped signal), 0.94 (3H, s, Me-18). **^13^C-NMR** (CDCl_3_, 75 MHz, ppm): δ 185.91 (C11), 183.40 (C14), 169.83 (7α-COCH3), 151.04 (C12), 150.04 (C9), 137.19 (C8), 124.76 (C13), 68.86 (C7), 67.06 (C6), 49.86 (C5), 42.39 (C3), 38.75 (C10), 38.55 (C1), 33.80 (C18), 24.28 (C15), 23.94 (C19), 21.60 (C20), 21.08 (7α-COCH3), 19.97 (C16), 19.84 (C17), 19.10 (C2).

**ParvD (3):** Orange powder, **^1^H-NMR** (CDCl_3_, 300 MHz, ppm): δ 7.93 (2H, d, H-2’ and H-6’), 6.96 (1H, d, *J* = 6.8 Hz, H-14), 6.88 (2H, d, H-3’ and H-5), 6.79 (1H, d, *J* = 6.9 Hz, H-7), 6.41 (1H, s, *J* = 12.5 Hz, H-6), 5.59 (1H, tt, *J* = 4.4 Hz, H-2β), 3.76 (1H, ddd, *J* = 11.4 Hz, H-1β), 3.15 (1H, m, H-15), 2.15 (1H, ddd, *J* = 4.4 Hz, H-3β), 1.74 (1H, dd, *J* = 13.0 Hz, H-1α), 1.64 (3H, s, Me-20), 1.56 (1H, dd, *J* = 11.4 Hz, H-3α), 1.42 (3H, s, Me-19), 1.29 (3H, s, Me-18), 1.18 (3H, d, *J* = 0.8 Hz, Me-16), 1.16 (3H, d, *J* = 2.4 Hz, Me-17). **^13^C-NMR** (CDCl_3_, 75 MHz, ppm): δ 178.24 (C12), 166.18 (C7’), 164.84 (C5), 160.58 (C4’), 146.50 (C11), 141.61 (C13), 139.30 (C7), 133.57 (C14), 131.89 (C2’ and C6’), 127.45 (C8), 127.17 (C9), 122.43 (C1’), 118.69 (C6), 115.23 (C3’ and C5’), 67.87 (C2), 45.06 (C3), 43.91 (C10), 38.58 (C4), 38.37 (C1), 33.03 (C18), 30.58 (C19), 26.52 (C15), 25.52 (C20), 21.84 (C16), 21.63 (C17)

**RoyBz (4):** Yellow quadrangular plates (EtOAc–*n*-pentane), ^1^**H-NMR** (CDCl_3_, 400 MHz, ppm): δ 8.15 (2H, dt, *J*2’,3’ = 7.6 Hz, *J*2’,4’ = 2.0 Hz, H–2’ and H–6’, 6–OBz), 7.99 (2H, dt, *J*2’,3’ = 7.2 Hz, *J*2’,4’ = 1.2 Hz, H-2’ and H–6’, 12–OBz), 7.69–7.40 (6H, complex signal, H–3’, H–4’, H–5’, 6– and 12–OBz), 5.90 (1H, dd, *J*7β,6α = 2.0 Hz, *J*7β,5α = 0.9 Hz, Hβ–7), 5.77 (1H, dd, *J6*α,7β *=* 2.0 Hz, *J*6α,5α = 1.6 Hz, Hα–6), 3.17 (1H, sept, *J*15,16(17) = 7.1 Hz, H–15), ~2.58 (1H, Hβ–1, overlapped signal), 2.10 (3H, *s*, OAc-7*α*), ~1.78 (1H, Hβ–2, overlapped signal), 1.77 (3H, s, Me-20), 1.69 (1H, br d, *J*5α,6α = 1.6 Hz, Hα–5), 1.58 (1H, H*α*–2, overlapped signal), 1.49 (1H, ddd, *J*3β,3*α* = 13.2 Hz, *J*3β,2α = 3.6 Hz, *J*3β,2β = 2.8 Hz, Hβ–3), 1.29 (1H, td, *J*1α,1β = *J*1α,2β = 13.2 Hz, *J*1α,2α = 3.8 Hz, H*α*–1), ~1.38 (1H, H*α*–3, overlapped signal), 1.21 (3H, d, *J*17,15 = 7.1 Hz, Me-17), 1.19 (3H, d, *J*16,15 = 7.1 Hz, Me-16), 1.06 (3H, s, Me-18), 0.99 (3H, *s*, Me-19). ^13^C **NMR** (CDCl_3_, 100 MHz, ppm): δ 185.33 (C-14, s); 180.00 (C-11, s); 168.13 (7*α*–OAc, s); 165.33 (C-7’, 6–OBz, s); 164.00 (C-7’, 12–OBz, s); 152.50 (C-9, s); 150.00 (C-12, s); 140.00 (C-13, s); 136.20 (C-8, s); 134.30 (C-4’, 12–OBz, d); 133.22 (C-4’, 6–OBz, s); 130.51 (C-3’ and C-5’, 12–OBz, d); 129.87 (C-3’ and C-5’, 6–OBz, d); 129.71 (C-1’, 6– OBz, s); 128.81 (C-2’ and C-6’, 12–OBz, d); 128.47 (C-2’ and C-6’, 6–OBz, d); 127.91 (C- 1’, 12–OBz, s); 68.43 (C-6, d); 65.25 (C-7, d); 49.31 (C-5, d); 42.48 (C-3, t); 38.77 (C-10, s); 38.37 (C-1, t); 33.76 (C-4, s); 33.33 (C-18, q); 25.16 (C-15, d); 23.21 (C-19, q); 22.20 (C-20, q); 20.84 (7α*-* OAc, q); 20.36 (C-16, q); 20.00 (C-17, q); 18.83 (C-2, t);

**RoyBzCl (5):** Yellow amorphous powder, **^1^H-NMR** (CDCl_3_, 400 MHz, ppm): δ 8.08 (2H, d, *J*o = 8.6 Hz, H–2’ and H–6’, 12–OBzCl), 7.92 (2H, d, *J*o = 8.6 Hz, H–2’ and H–6’, 6–OBzCl), 7.51 (2H, d, *J*o = 8.6 Hz, H–3’ and H–5’, 12–OBzCl), 7.39 (2H, d, *J*o = 8.6 Hz, H–3’ and H–5’, 6–OBzCl), 5.88 (1H, d, *J*7β,6α = 1.6 Hz, Hβ–7), 5.76 (1H, t, *J*6α,7β *= J*6α,5α = 1.6 Hz, Hα–6), 3.17 (1H, sept, *J* 15,16(17) = 7.0 Hz, H–15), 2.58 (1H, Hβ–1, overlapped signal), 2.10 (3H, s, 7α–OAc), 1.79 (1H, qt, *J*2β,1α = *J*2β,2α = *J*2β,3α = 14.0 Hz, *J*2β,1β = *J*2β,3β = 3.7 Hz, Hβ–2), 1.74 (3H, s, Me-20), 1.70 (1H, d, *J*5α,6α = 1.6 Hz, Hα–5), 1.60 (1H, *dquint*, *J*2α,*2*β = 14.0 Hz, *J*2*α*,1*α* = *J*2α,1β = *J*2*α*,3*α* = *J*2*α*,3β = 3.6 Hz, H*α*–2); 1.48 (1H, dtd, *J*3β,3α = 13.4 Hz, *J*3*β,*2β = 3.7 Hz, *J*3β,2α = 3.6 Hz, *J*3β,1β = 1.0 Hz, Hβ–3), 1.30 (1H, Hα–1, overlapped signal), 1.28 (1H, H*α*–3, overlapped signal), 1.21 (3H, d, *J*16,15 = 7.0 Hz, Me-16), 1.19 (3H, d, *J*17,15 = 7.0 Hz, Me-17), 1.06 (3H, s, Me-18), 0.97 (3H, s, Me-19). **^13^C NMR** (CDCl_3_, 100 MHz, ppm): δ 185.21 (C-14, s); 179.70 (C-11, s); 168.13 (OAc-7α*,* s); 164.52 (C-7’, 6–OBzCl, s), 163.19 (C-7’, 12–OBzCl, s); 152.40 (C-9, s); 149.50 (C-12, s); 141.02 (C-4’, 12–OBzCl, s); 139.80 (C-13, s); 139.79 (C-4’, 6–OBzCl, s); 135.67 (C-8, s); 131.84 (C-2’ and C-6’, 12–OBzCl, d); 131.22 (C-2’ and C-6’, 6–OBzCl, d); 129.24 (C-3’ and C-5’, 12–OBzCl, d); 128.88 (C-3’ and C-5’, 6–OBzCl, d); 128.14 (C-1’, 6–OBzCl, s); 126.36 (C-1’, 12–OBzCl, s); 68.72 (C-6, d); 65.19 (C-7, d); 49.30 (C-5, d); 42.47 (C-3, t); 38.78 (C-10, s); 38.37 (C-1, t); 33.77 (C-4, s); 33.29 (C-18, q); 25.23 (C-15, d); 23.20 (C-19, q); 21.23 (C-20, q); 20.37 (C-16, q); 20.21 (C-17, q); 18.81 (C-2, t).

**RoyPr2 (6):** Yellow rectangular plates, (EtOAc–*n*-pentane), **^1^H-NMR** (CDCl_3_, 400 MHz, ppm): δ 5.68 (1H, dd, *J*7β,6α = 2.0 Hz, *J*7β,5α = 0.5 Hz, Hβ–7), 5.49 (1H, br dd, *J*6α,7β = 2.0 Hz, *J*6α,5α = 1.6 Hz, Hα–6), 3.09 (1H, sept, *J*15,16(17) = 7.1 Hz, H– 15), 2.66 (1H, dq, *J*2’A,2’B = 17.2 Hz, *J*2’A,3’ = 7.6 Hz, H2’A–12), 2.61 (1H, dq, *J*2’B,2’A = 17.2 Hz, *J*2’B,3’ = 7.6 Hz, H2’B–12), 2.51 1H, (br d, *J*1α,1β = 12.4 Hz, Hβ–1), 2.32 (1H, dq, *J*2’A,2’B = 16.8 Hz, *J*2’A,3’ = 7.6 Hz, HA–2’), 2.25 (1H, q, *J*2’B,2’A = 16.8 Hz, *J*2’B,3’ = 7.6 Hz, HB–2’), 2.04 (3H, s, OAc-7*α*), 1.77 (1H, dddt, *J*2β,2α = 14.4 Hz, *J*2β,1α = *J*2β,3α = 13.8 Hz, *J*2β,1β = *J*2β,3β = 3.6 Hz, Hβ–2), 1.59 (3H, s, Me-20), 1.55 (1H, dquint, *J*2α,2β = 14.4 Hz, *J*2α,1α = *J*2α,1β = *J*2α,3α = *J*2α,3β = 3.6 Hz, Hα–2), 1.53 (1H, dd, *J*5α,6α = 1.6 Hz, *J*5α,7β = 0.5 Hz, Hα–5), 1.44 (1H, dtd, *J*3β,3α = 13.8 Hz, *J*3β,2α = *J*3β,2β = 3.6 Hz, *J*1β,3β = 1.6 Hz, Hβ–3), 1.27 (3H, t, *J*3’’,2’’A = *J*3’’,2’’B = 7.6 Hz, Me-3’’), 1.24 (1H, Hα–1, overlapped signal), 1.23 (1H, Hα–3, overlapped signal), 1.17 (3H, d, *J*16,15 = 7.1 Hz, Me- 16), 1.16 (3H, d, *J*17,15 = 7.1 Hz, Me-17), 1.11 (1H, t, *J*3’,2’A = *J*3’,2’B = 7.6 Hz, H-3’), 0.98 (3H, s, Me-18), 0.96 (3H, s, Me-19); ^13^**C NMR** (CDCl_3_, 100 MHz, ppm): δ 185.37 (C-14, s); 179.67 (C-11, s); 172.50 (C-1’, s); 171.73 (C-1’’, s), 168.15 (OAc-7*α*, s); 152.22 (C-9, s); 149.28 (C-12, s); 139.42 (C-13, s); 135.67 (C-8, s); 67.24 (C-6, d); 65.25 (C-7, d); 48.95 (C-5, d); 42.39 (C-3, t); 38.93 (C-10, s); 38.31 (C-1, t); 33.58 (C-4, s); 33.18 (C-18, q); 27.81 (C-2’, t), 27.21 (C-2’’, t); 25.17 (C- 15, d); 23.03 (C-19, q); 21.44 (C-20, q); 21.27 (OAc-7α, q); 20.17 (C-16, q); 20.19 (C-17, q); 18.78 (C-2, t), 8.86 (C-3’’, q); 8.80 (C-3’, q).

**DihydroxyRoy (7):** Yellow needles, **^1^H NMR** (CDCl_3_, 400 MHz, ppm): δ 7.27 (1H, s, OH-12), 7.25 (1H, s, OH-6β), 4.51 (1H, dd, *J =* 3.3 Hz, *J*7β,6α = 2.0 Hz, H-7β), 4.45 (1H, dd, *J*6α,5α = 4.0 Hz, *J*6α,7β = 2.0 Hz, H-6α), 3.16 (1H, sept, *J*15,16(17) = 7.1 Hz, H-15), 2.93 (1H, d, *J*OH,7β *=* 3.3 Hz, OH-7α), 2.59 (1H, dddd, *J*1β,1α = 12.8 Hz, *J*1β,2α = 3.5 Hz, *J*1β,2β = 3.5 Hz, *J*1β,3β(W) = 1.3 Hz, H-1β), 1.83 (1H, ddddd, *J*2β,1α = 13.4 Hz, *J*2β,1β = 3.5 Hz, *J*2β,2α = 13.9 Hz, *J*2β,3α = 13.4 Hz, *J*2β,3β = 3.4 Hz, H-2β), 1.60 (3H, s, Me-20), ~1.56 (1H, *J*2α,1α = 3.8 Hz, *J*2α,1β = 3.5 Hz, *J*2α,2β = 13.9 Hz, *J*2α,3α = 4.1 Hz, *J*2α,3β = 3.4 Hz, H-2α, overlapped signal), 1.47 (1H, dddd, *J*3,2α = 3.4 Hz, *J*3β,2β = 3.4 Hz, *J*3β,3α = 13.4 Hz, *J*3β(W),1β = 1.3 Hz, H-3β), 1.40 (1H, d, *J*5α,6α = 4.0 Hz, H-5α), 1.25 (3H, s, Me-19), 1.22 (1H, ddd, *J*3α,2α = 4.1 Hz, *J*3α,2β = 13.4 Hz, *J*3α,3β = 13.4 Hz, H-3α), 1.22 (3H, d, J16(17),15 = 7.1 Hz, Me-16), 1.21 (3H, d, *J*_17,15_ = 7.1 Hz, Me-17), 1.18 (1H, ddd, *J*_1α,1β_ = 12.8 Hz, *J*_1α,2α_ = 3.8 Hz, *J*_1α,2β_ = 13.4 Hz, H-1α), 1.04 (3H, s, Me-18). ^13^C NMR (100 MHz, CDCl_3_, ppm): δ 38.5 (C-1, t); 19.0 (C-2, t); 42.3 (C-3, t); 33.8 (C-4, s); 49.5 (C-5, d); 69.4 (C-6, d); 69.2 (C-7, d); 141.0 (C-8, s); 147.5 (C-9, s); 38.6 (C-10, s); 183.5 (C-11, s); 151.1 (C-12, s); 124.3 (C-13, s); 189.1 (C-14, s); 24.3 (C-15, d); 19.9# (C-16, q); 19.8# (C-17, q); 33.5 (C-18, q); 24.0 (C-19, q); 21.6 (C-20, q).

### 4.2. Cytotoxicity Assay

In order to screen the cytotoxicity of the isolated compounds **1**-**3**, three breast cancer cell lines representing three major classes of breast cancer were selected: MCF-7 cell line expressing estrogen receptors, SkBr3 expressing high levels of Her2^NEU^, and SUM159 cell line negative for both hormone receptors and for Her2. The named cell lines were cultivated in DMEM with 10% fetal calf serum. Additionally, SUM159 cells were also cultivated under cancer stem cell-inducing conditions in special media with 10 ng/mL basic fibroblast growth factor (bFGF), 20 ng/mL epidermal growth factor (EGF), 5 μg/mL insulin (all three from Peprotech, New York, USA), and 20 μL/mL B27 supplement (Invitrogen), and are referred to as SUM159 sphere. 

### 4.3. Cell Viability Assay

Cells grow in this media from spheres and increase the number of cells expressing markers of cancer stem cells CD44^+^/CD24^-^/ESA^low^. All cell lines were cultivated at 37 °C in a humidified atmosphere with 5% CO_2_. For the MTT viability assay, cells were harvested, counted, and seeded at 10,000 cells/well. Cells were left for 24h to attach and then were treated with 1, 10, and 100 µM solutions of **1**, **2**, and **3**. Cells were left for 24 h and then were assayed for viability with the EZ4U MTT assay (Biomedica, Austria) according to manufacturer’s instructions. Briefly, 20 µL of colorless dye was added to each well. Mitochondria of the living cells oxidize the dye to a yellow-colored formazan derivative, which is then assayed on the plate reader at 450 nm with a reference wavelength of 620 nm. All experiments were performed in biological duplicate and technical triplicates and were expressed as mean ± SDs. Statistical analysis between groups was performed by two-way ANOVA with Tukey’s multiple comparisons test. *p* values < 0.05 were considered statistically significant. Statistical analyses were performed using the GraphPrism 7.0 software [43].

### 4.4. Ligands

The structure for the ligands was drawn in ChemDraw 17.0 [44] and converted to structure mol files, energy minimized with MacroModel [45], with two different conformers generated for **2**: One with the terminal cyclohexane ring in the chair conformation, another with the terminal cyclohexane ring in the half-chair conformation. The chair ring conformation was kept for further studies giving better results than the other conformer. Ligands phorbol 12-myristate 13-acetate (PMA) and arachidonic acid (ARA) were also used as controls. For docking in AutoDock Vina [46], ligands were prepared in ADT Tools 1.5.6 [47]. 

### 4.5. Sequence Alignment

The protein sequences for PKC isoforms *α*, *β*, *i*, *δ*, *ε*, *ζ*, *ι*, and *θ* were downloaded from the Uniprot database [48] and aligned with Clustal 2.1 [49].

### 4.6. Docking

Two programs with different scoring functions and optimization algorithms were used: AutoDock Vina [46] and Glide XP (extra precision) v2018-2 [50] with Molecular Mechanics Generalize Born/Surface Area (MMGBSA) post-processing. The protein crystal structures of human PKC isoforms *α*, *β*, *ι*, and *θ*, with codes 4RA4, 2I0E, 3ZH8, and 5F9E, respectively, were downloaded from RCSB Protein Data Bank [37] and preprocessed with ADT Tools 1.5.6 for calculating protonation states and to add hydrogen atoms [47]. Crystallographic waters were not included in the structures for docking. The co-crystallized ligands were also self-docked into their respective binding sites in order to calculate a reference value for a positive control for the docking calculations. 

### 4.7. Vina

The grid box with dimensions 24 × 24 × 24 Å was set in the center of the protein binding site, spanning all the amino acid residues involved in binding based on the coordinates of co-crystalized ligands. The exhaustiveness was set to 50. Ten runs were made for each docking calculation.

### 4.8. Glide

An inner grid box with a size of 25 Å per side was used as in previous procedures [51,52,53]. The extra precision XP scoring function was employed. Parameter LIG_VCUT was set to 0.8 (default value) to scale ligand van der Waals radii by 80% for those atoms with a partial charge smaller than 0.15 in order to reduce steric clashes with the protein. The Virtual Screening protocol included a first screen with HTVS, retaining all good-scoring poses, through to scoring with SP, again retaining all good-scoring poses, then docking with the XP (extra precision) scoring function, and final post-processing by calculating the MMGBSA energies for the final binding poses.

## 5. Conclusions

Subtle changes in the binding site of each PKC isoform change the predicted interaction profiles of the ligands. Subtle changes in the royleanone substitution patterns, such as a double substitution only with non-substituted phenyls, can increase certain predicted subtype specificity as is seen in the increased interaction score with PKC*δ* isoform for RoyBz (**4**). On the other hand, a hydroxybenzoate substituent on position four of the royleanone core can provide a better binding pose in PKC*ι*, as seen for ParvD (**3**),. DeRoy (**1**) and ParvD (**3**) are the strongest predicted binders to all isoforms computationally and to all breast cancer types experimentally, perhaps due to their interaction with the conserved Lys in the catalytic site. They may also interact with hydrophobic residues in the hydrophobic motif of atypical PKCs (isoform *ι*/*λ*/PKM*ζ*), which may grant specificity to those isoforms. Their relatively small size and deep and low exposure to solvent when in their bound conformation may indicate binding advantages over other compounds. ParvD (**3**) has the strongest predicted interaction profile for all isoforms. Studying the predicted structure–activity relationships, such as those between promising royleanone compounds and different subtypes of PKC and types of cancer cells, such as the hard to treat triple-negative cancer stem cells or tumor-initiating cells, can provide further indications to guide the design of compounds that can inhibit particular PKC subtypes, or all of them, as well as being active against aggressive triple-negative cancer cells. An interesting new compound for all isoforms can be proposed by substituting a hydroxybenzoate on position four of the royleanone core of DeRoy (**1**). Perhaps, the introduction of benzoate groups of the type in RoyBz (**4**) onto other royleanones may increase the specificity for PKC*δ*.

## Figures and Tables

**Table 1:** Percent identity matrix from the sequence alignment of protein isoforms.

Uniprot, Isoform	*ζ*	*Ι*	*δ*	*θ*	*ε*	*α*	*β*
sp|P41743|KPCI_HUMAN, *ζ*	100	72.81	36.82	36.86	44.78	43.57	43.48
sp|P41743|KPCI_HUMAN, *ι*	72.81	100	36.14	36.52	43.91	45.36	44.21
sp|Q05655|KPCD_HUMAN, *δ*	36.82	36.14	100	64.89	43.88	47.61	48.99
sp|Q04759|KPCT_HUMAN, *θ*	36.86	36.52	64.89	100	43.31	48.51	47.64
sp|Q02156|KPCE_HUMAN, *ε*	44.78	43.91	43.88	43.31	100	52.9	53.14
sp|P17252|KPCA_HUMAN, *α*	43.57	45.36	47.61	48.51	52.9	100	79.01
sp|P05771|KPCB_HUMAN, *β*	43.48	44.21	48.99	47.64	53.14	79.01	100

**Table 2:** Calculated docking scores for compounds against PKC isoforms. All values in kcal/mol.

Compound	5f9e (Isoform *θ*)			2i0e (Isoform *β*II)			3zh8 (Isoform *ι*)			4ra4 (Isoform α)			1ptr (Isoform *δ*)		
	Vina	Glide XP	MMGBSA	Vina	Glide XP	MMGBSA	Vina	Glide XP	MMGBSA	Vina	Glide XP	MMGBSA	Vina	Glide XP	MMGBSA
PMA	−7.4			−8.0	−4.84	−40.13	−7.2			−6.4			−4.7	−4.41	−42.12
ARA	−6.3			−7.8	−1.00	−40.95	−6.5			−5.6			−4.4	−1.85	−18.56
5VS1001 (5f9e co-cryst.)	−10.5	−7.2	−40.48												
PDS 902 (2i0e co-cryst.)				–11.0	−10.02	−56.23									
C581582 (3zh8 co-cryst.)							−9.9	−8.0	−58.56						
3KZ701 (4ra4 co-cryst.)										−10.4	−10.0	0			
PRB3 (1ptr co-cryst.)													−6.3	−4.25	−27.00
**1** (DeRoy)	–9.3	−5.8	−44.29	−12.0	−6.21	−30.81	−8.4	−5.6	−36.23	−8.4	−6.7	0	−6.2	−4.13	−29.39
**2** (Roy)	–9			−10.4			−8.8			−8			−6.7		
**3** (ParvD)	–9.8	−2.0		−12.0	−5.84		−9.8	−6.91		−9.3	−4.84		−8.4	−4.68	
**4** (RoyBz)	–9.3			−9.4			−9.0			−8.7			−6.9		
**5** (RoyBzCl)	–8.8			−9.8			−9.4			−8.4			−6.7		
**6** (RoyPr_2_)	–8.7			−7.9			−7.4			−7.5			−6.3		
**7** (DihidroxyRoy)	–8.3			−10.5			−8.7			−7.6			−7.9		

**Table 3:** PKC isoform binding site amino acids and corresponding interactions.

Compounds	PKC Isoform			
	*α*	*β* *I*	*ι*	*θ*
**DeRoy (1)**	Met 417 (L), Ala 480 (L), Met 470 (L), Lys 368 (H), Val 353 (L), Leu 345 (L)	Met 473 (L), Ala 483 (L), Phe 485 (L), Leu 394 (L), Phe 353 (L), Val 356 (L), Lys 371 (L), Phe 418 (L), Met 420 (L), Ala 369 (L), Leu 348 (L)	Leu 376 (L), Thr 386 (R), Val 307 (L), Phe 297 (L), Ile 323 (L), Lys 274 (L), Val 259 (L), Ile 251 (L)	Leu 511 (L), Ala 521 (L), Met 458 (L), Lys 409 (H), Val 394 (H), Phe 391 (R)
**Roy (2)**	Val 420 (H), Lys 368 (L), Ala 366 (L), Met 417 (L), Val 353 (L), Leu 345 (H, L)	Met 473 (L), Ala 483 (L), Phe 353 (L), Met 420 (L), Lys 371 (L), Val 356 (L)	Asp 373 (H), Ile 323 (L)	Leu 511 (L), Asn 509 (L), Ala 521 (L), Met 458 (L), Lys 409 (L), Val 394 (L), Leu 386 (L)
**ParvD (3)**	Met 470 (L), Val 353 (L), Ala 366 (L), Leu 345 (L), Met 417 (R, L), Lys 368 (L), Leu 391 (L), Ala 480 (L)	Met 473 (L), Tyr 422 (L), Leu 348 (L), Val 356 (L), Ala 483 (L), Ala 369 (L), Asn 471 (H), Phe 485 (L), Leu 394 (L), Lys 371 (L)	Ile 251 (L), Val 259 (L), Leu 376 (L), Tyr 325 (H), Val 259 (L), Thr 386 (L), Lys 274 (L), Ile 323 (L), Val 307 (L), Phe 297 (L)	Leu 511 (L), Ala 521 (L), Met 458 (L), Ala 407 (L), Val 394 (L), Phe 391 (L)
**RoyBz (4)**	Asp 424 (H), Ala 366 (L), Val 353 (L), Met 417 (L), Lys 368 (L), Ala 480 (L)	Leu 348 (L), Met 473 (L), Val 356 (L), Phe 353 (L), Lys 371 (L), Met 420 (L), Ala 483 (L)	Phe 333 (L, R), Asp 330 (H), Ile 251 (L), Leu 376 (L), Val 259 (L), Thr 386 (R), Ala 272 (L), Ile 323 (L), Val 307 (L)	Gly 464 (L), Phe 391 (L), Val 394 (L), Ala 407 (L), Met 458 (L), Ala 521 (L), Asp 522 (L), Lys 409 (H)
**RoyBzCl (5)**	Asp 424 (H), Gly 423 (L), Met 343 (R, L), Val 353 (L), Phe 350 (L), Lys 368 (L), Met 417 (L), Ala 480 (L)	Met 473 (L), Ala (483), Leu 394 (L), Met 420 (R, L), Lys 371 (L), Val 356 (L), Phe 353 (L), Leu 348 (L)	Phe 333 (R), Asp 330 (H), Thr 386 (R), Val 307 (L), Ile 323 (R), Ala 272 (R), Val 259 (R, L), Ile 251 (L), Arg 253 (R, L)	Leu 511 (L), Ala 521 (L), Lys 506 (L), Phe 391 (R), Val 394 (L), Leu 386 (R), Tyr 460 (L)
**RoyPr_2_ (6)**	Asp 424 (H), Met 470 (L), Val 420 (L), Met 417 (L), Ala 366 (L), Val 353 (L)	Ala 483 (L), Phe 383 (L), Lys 371 (L), Val 356 (L), Leu 348 (L)	Thr 386 (H), Leu 376 (L), Ile 251 (L), Val 259 (L), Ala 257 (L)	Leu 511 (L), Ala 521 (L), Met 458 (L), Lys 409 (H, L), Ala 407 (L), Val 394 (L), Phe 391 (L)
**DihydroxyRoy (7)**	Met 470 (L), Val 420 (H), Met 417 (L), Lys 368 (L), Leu 345 (H, L)	Phe 353 (L), Leu 348 (L), Val 356 (L), Lys 371 (L), Met 420 (R, L), Leu 394 (L), Phe 485 (L), Ala 483 (L)	Asp 373, Val 259, Lys 274, Ala 272, Ile 323	Leu 511 (L), Ala 521 (L), Phe 523 (L), Leu 432 (L), Met 458 (L), Lys 409 (L), Val 394 (L), Leu 386 (L), Phe 391 (R)

H—Hydrophobic interaction; L—Hydrophilic interaction; R—aromatic interaction.

**Table 4:** Corresponding amino acid residues in different PKC isoforms.

PKC*α*	PKC *β*I	PKC*ι*	PKC*θ*
Met 470	Met 473	Leu 376	Leu 511
Ala 480	Ala 483	Thr 386	Ala 521
Thr 401	Thr 404	Val 307	Thr 442
Met 417	Met 420	Ile 323	Met 458
Lys 368	Lys 371	Lys 274	Lys 409
Val 353	Val 356	Val 259	Val 394
Leu 345	Leu 348	Ile 251	Leu 386

**Table 5:** Compound octanol/water partition (log*P*) values and solvent-accessible surface area for docked poses in PKCα isoform.

Compound	Total Solvent Accessible Area (Å^2^)	Solved Exposed Area in Docked Pose (Å^2^)	Exposed Surface Ratio %	log*P*
DeRoy (1)	268.47	142.36	53.02	4.53
Roy (2)	318.47	209.29	65.71	2.65
ParvD (3)	171.75	355.37	48.12	5.64
RoyBz (4)	469.79	260.01	55.35	7.88
RoyBzCl (5)	504.92	323.30	64.03	8.8
RoyPr_2_ (6)	403.77	207.97	51.50	4.87
DihidroxyRoy (7)	285.37	165.58	58.02	2.52
